# Age-Specific Average Head Template for Typically Developing 6-Month-Old Infants

**DOI:** 10.1371/journal.pone.0073821

**Published:** 2013-09-12

**Authors:** Lisa F. Akiyama, Todd R. Richards, Toshiaki Imada, Stephen R. Dager, Liv Wroblewski, Patricia K. Kuhl

**Affiliations:** 1 University Of Washington, Institute for Learning and Brain Sciences, Seattle, Washington, United States of America; 2 Department of Radiology, University of Washington, Seattle, Washington, United States of America; 3 Autism Center, University of Washington, Seattle, Washington, United States of America; 4 Department of Bioengineering, University of Washington, Seattle, Washington, United States of America; Biomedical Imaging Lab, Agency for Science, Singapore

## Abstract

Due to the rapid anatomical changes that occur within the brain structure in early human development and the significant differences between infant brains and the widely used standard adult templates, it becomes increasingly important to utilize appropriate age- and population-specific average templates when analyzing infant neuroimaging data. In this study we created a new and highly detailed age-specific unbiased average head template in a standard MNI152-like infant coordinate system for healthy, typically developing 6-month-old infants by performing linear normalization, diffeomorphic normalization and iterative averaging processing on 60 subjects’ structural images. The resulting age-specific average templates in a standard MNI152-like infant coordinate system demonstrate sharper anatomical detail and clarity compared to existing infant average templates and successfully retains the average head size of the 6-month-old infant. An example usage of the average infant templates transforms magnetoencephalography (MEG) estimated activity locations from MEG’s subject-specific head coordinate space to the standard MNI152-like infant coordinate space. We also created a new atlas that reflects the true 6-month-old infant brain anatomy. Average templates and atlas are publicly available on our website (http://ilabs.washington.edu/6-m-templates-atlas).

## Introduction

A common concern in infant neuroimaging analyses is spatial normalization of the infant brain structure and the problem of relating functional activations in the brain to a common reference frame. Many have questioned the usage of the widely used standard average adult brain templates, such as MNI305 and MNI152 [1]-[6] as reference templates for infant and pediatric populations due to significant morphometric differences [7]-[13]. Warping pediatric brains to adult reference data requires substantial deformation, carries the risk of misclassifying the brain tissue, and can introduce errors as large as a full centimeter [12], [14]. Thus, it is preferable to avoid the standard adult reference data when spatially normalizing data from other populations, such as infants and children, in order to optimize neuroimaging analysis. Indeed, normalization to custom templates has been reported to improve localization accuracy, lead to unbiased statistics, and produce more biologically likely results [15]–[19].

Creation of standard average pediatric brain templates is complicated by significant developmental variability in brain anatomy and morphometry within the infant and pediatric populations. The whole brain, frontal lobe and temporal lobe volumes experience growth spurts during the first 2 years of life [20]. Phenomenal developmental growth occurs in the first year of life: 2 to 4 week olds have ∼40% and 1 year olds have ∼72% of adult cerebral volume [21], and whole brain volume increases from ∼645 cm^3^ to ∼970 cm^3^ from 3 to 13 months of age [22]. More specifically, brain regions develop asynchronously at different rates. By 12 months of age, the cerebrum, putamen, globus pallidus and cerebellar hemisphere reach up to ∼70% of the volume of 7 to 11 year olds, whereas the hippocampus and amygdala only attain ∼50% of the volume of 7 to 11 year olds, exemplifying that brain sub regions also develop at different speeds [22]. Due to these dramatic developmental changes that occur on the scale of months during the first year, it is crucial to have truly age-specific reference templates when performing spatial normalization.

In addition to age- and population-specific standard templates, it is desirable to have an appropriate stereotaxic coordinate system that is specialized for the significantly different head size represented by infant populations. Having a standard infant-specific coordinate system will enable us to make intra-population and inter-population statistical comparisons and report functional activation areas using standard coordinates, similar to reporting brain activity locations using standard MNI350/MNI152 coordinates in adult studies. Recently a uniform infant-specific coordinate system that is compatible with the commonly used adult MNI152 coordinate system was created. This stereotaxic coordinate system is a scaled down version of the MNI152 coordinate system that represents the average head size of infants aged 5 to 8 months [2]. Fonov et al. [2] successfully created and publicly released an infant average template [referred to as the NIHPD5–8m template] that is in an MNI152-like infant coordinate system [referred to as iMNI] created by averaging 1.5 Tesla (T) magnetic resonance (MR) images (2D scans; voxel size 1×1×3 mm) of infants ages 5–8 months (*n* = 40) from the NIH-funded MRI Study of Normal Brain Development, Pediatric MRI Data Repository (NIHPD) [23], [24]. Sanchez et al. [25] constructed infant and pediatric templates at 1.5 month age increments for ages between 2 weeks and 4 years, including 6 month old templates created by averaging NIHPD 1.5T MR images, 3T MR images or combined 1.5T and 3T MR images. Nevertheless, concerns remain that average templates created from a small sample such as Fonov et al. [2] and Sanchez et al. [25] may not capture the wide range of variance within the specific population and thus could potentially introduce unwanted biases.

The purpose of the present study was to construct a new, high resolution, age- and population-specific average template for healthy 6-month-old infants that can be utilized to perform accurate infant spatial normalization and statistical comparison in various neuroimaging studies. We obtained a large number of brain images of healthy, typically developing 6-month-old infants representative of the U.S. population in regards to race/ethnicity. A diffeomorphic normalization algorithm [26]-[28] and iterative averaging method that uses a shape and appearance averaging technique [29], [30] were employed to produce this age-representative standard head template for 6-month-old infants. Similar template creating techniques were employed by Sanchez et al. [25]. We illustrate an example usage of this average template to convert MEG estimated activity locations in MEG subject-specific head coordinate system to the iMNI infant coordinate system. Furthermore, a 6-month-old brain atlas that is compatible with this template was created. The resulting template will be contributed to the scientific community for various neuroimaging analysis purposes in the study of 6-month-old infants.

## Methods

### Ethics Statement

The paper reports data from human subjects, and ethical approval was obtained from the University of Washington Human Subjects Division and Seattle Children’s Institutional Review Board. Written informed consent was obtained from parents or legal guardians of all participants according to the principles explained in the Declaration of Helsinki, and the rights of these participants were protected.

### Subjects

T1 MR images of 6-month-old (mean = 204.0±12.2 days, median = 202.5 days, range = 177–230 days) infants (*n* = 60; 29 males, 31 females) were acquired (Table 1). All subjects were healthy, typically developing full term infants (more than 36 weeks gestational age at birth) with no obvious congenital, neurological or physical abnormalities or impairments. Subjects had uneventful pre- and peri-natal circumstances and weighed more than 2,200 g at birth.

**Table 1:** Demographics of the subjects included in the average head template.

Scan Site	Tesla	Total number of subjects (female/male)	Mean Age (days)±standard deviation	Median Age (days)	Age Range (days)
University of Washington	1.5T	27 (13/14)	206.8±12.1	204	187–230
Seattle Children's Hospital	3T	33 (18/15)	202.3±12.5	200	177–225
** Overall**		**60 (31/29)**	**204.0±12.2**	**202.5**	**177–230**

### Image Acquisition

Subjects were not sedated and scans were performed during natural sleep. Before the scans, infants were well wrapped in blankets and earmuffs were securely placed to protect the ears. High-resolution T1-weighted anatomic images were acquired from 1.5T (*n* = 27; 14 males, 13 females) and 3T (*n* = 33; 15 males, 18 females) scanners from two sites. At the University of Washington, sagittal images (1.0 mm slice thickness) were acquired from GE Signa 1.5T (version 5.8) (General Electric, Milwaukee, WI, USA) using a 3D fast spoiled gradient echo pulse sequence. Imaging parameters were as follows: repetition time (TR) 11.1 ms, echo time (TE) 2.2 ms, flip angle 25°, field of view (FOV) 24 cm, voxel size 0.94×0.94×1 mm and reconstructed matrix size 256×256×124 mm. The entire acquisition time was 4 min 36 s. At Seattle Children’s Hospital, anatomical T1-weighted 3D images were acquired from a Siemens 3T TRIOTIM scanner (Siemens, Erlangen, Germany) using a gradient echo with inversion pulse sequence. The following parameters were specified: scanner software version Syngo MR B17, TR 2400 ms, TE 3.16 ms, flip angle 8°, FOV 256×224×160 mm, voxel size 1×1×1 mm, acquisition matrix 256×224×160 mm and inversion time 1200 ms. The entire acquisition time was 6 min 13 s.

### Average Template Creation

#### Image Pre-processing

**Figure 1:**
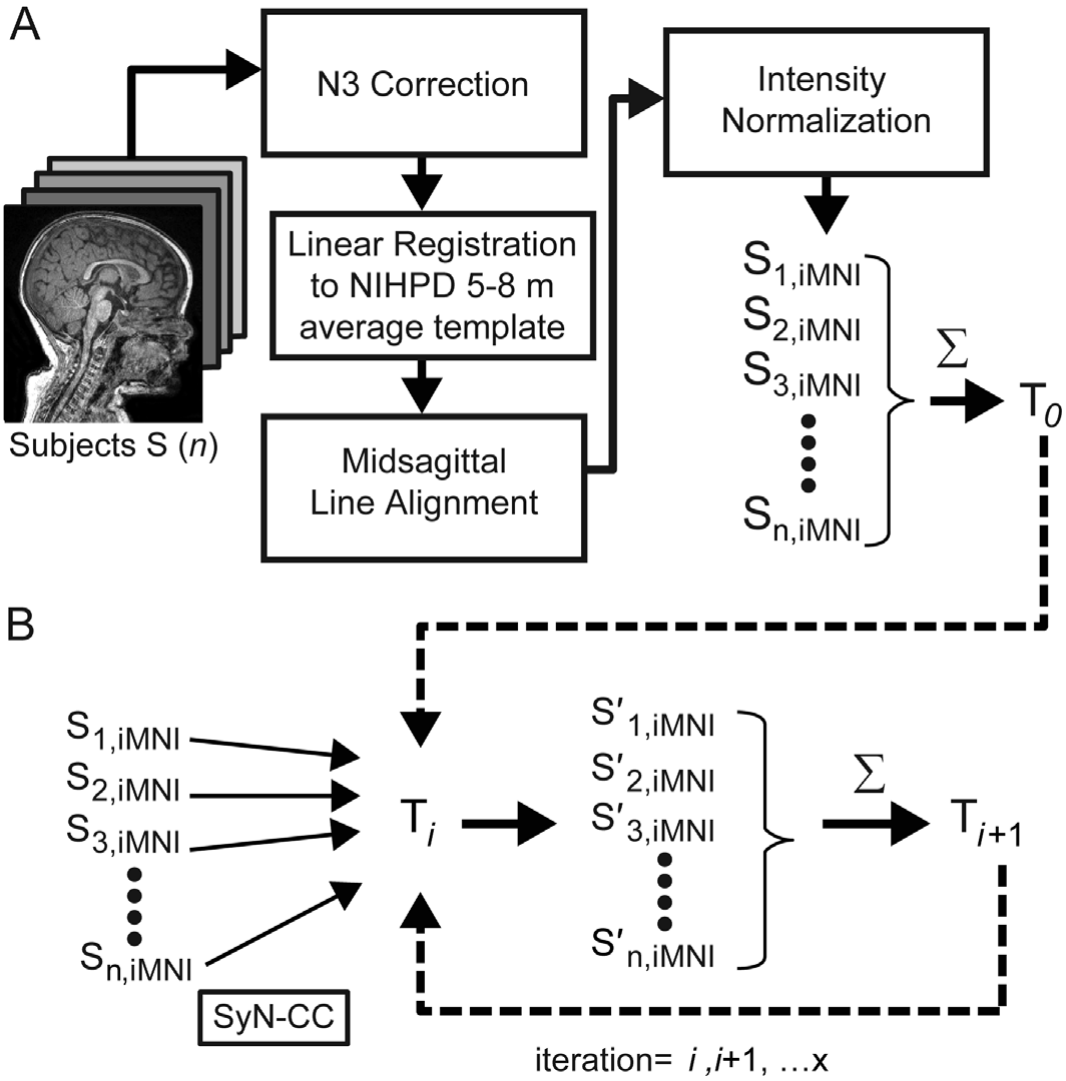
Average template creation. General overview of the pre-processing, linear registration, diffeomorphic normalization and iterative averaging steps involved in creating average template. (A) Pre-processing and rigid linear registration. (B) Diffeomorphic normalization and iterative averaging. These processes were performed using Advanced Normalization Tools (ANTS) [26]–[28].

All average template creation steps are outlined in Figure 1. Each subject’s image (S_n_) was corrected for intensity inhomogeneity using the N3 intensity non-uniformity correction algorithm [31], using tools from the Medical Image NetCDF [32] image-processing framework developed at the Montreal Neurological Institute (http://www.bic.mni.mcgill.ca/ServicesSoftware). Next, midsagittal line alignment was performed automatically (Medical Image Processing, Analysis and Visualization; MIPAV) [33], followed by manual alignment along the anterior commissure and posterior commissure (AC-PC) plane to correct for different head positions in the scanner (FMRIB’s Software Library (FSL) Nudge tool) [34].

#### Linear Registration

Pre-processed images were linearly registered using 6 parameters (rigid registration; rotation and translation only) to the NIHPD5–8m template in order to retain the 6-month-old infant brain size in our templates. Rigid registration was performed using MINC’s bestlinreg registration tool [35], [36] in order to preserve the unique age- and population-specific head size, shape and volume. Later, all linearly registered images (S_n,iMNI_) were averaged to create the initial average template (T_0_).

#### Nonlinear registration and iterative averaging

The symmetric diffeomorphic image normalization algorithm (SyN) provided through the Advanced Normalization Tools (ANTS) and iterative averaging method were implemented in creating a population-specific, unbiased average head template [26]. SyN, which has been evaluated as one the most reliable and well-performing nonlinear registration algorithms currently available [27]; [37], was chosen for its ability to capture large deformations while preserving the underlying regional anatomy [26], [27]. First, the intensity of each image was rescaled to be in the range of 0–10 by using a single linear histogram scaling [38]. Image information of both the input data and the reference data are then fed into the mapping optimization process and transformations are equally distributed to both the input and reference data, thus data are symmetrically normalized [26], [27]; the normalization process is unbiased towards the input data and the reference data. SyN functions use an optimization strategy based on minimizing the shape and appearance distances between the input data and reference data. Furthermore, cross-correlation (CC), a similarity metric commonly used for intramodality registration that maximizes the similarity of regional intensity patterns, was specified and included when performing the SyN gradient descent. That is, SyN was instructed to compute diffeomorphic mappings that maximize/optimize the CC between the input data and the reference data. It has been reported that SyN with the CC metric (SyN-CC) performs well even in conditions in which complex intensity relationships exist between the input data and the reference data and outperforms other numerous top performing algorithms [27], [39]).

SyN multi-resolution optimization sets maximum iterations of 100 over three resolutions (coarsest 1 mmx2*^n^*, next coarsest 1 mmx2*^n^* ^−1^, and full resolution 1 mmx2*^n^* ^−2^), where the number of levels in the multi-resolution Gaussian pyramid (*n*) is 3. This setting was chosen since three levels are typically used for 1 mm^3^ T1 MR images [28]. A gradient descent step size of 0.25 was specified for SyN gradient descent.

Template construction was optimized by minimizing shape and appearance distances [28]. Each S_n,iMNI_ was warped to the current average template (T*_i_*), initially T_0_, using SyN-CC (Figure 1b). Next, the warped scans, S′_n,iMNI_, were averaged, creating a new average template (T*_i_*_+1_). These steps were iterated (*x* times) by updating T*_i_*, the reference, current average template in the SyN-CC step, continuously until convergence of the average template was reached.

#### Evaluation of the average templates

Distinct templates were created from 1.5T images *(n = *27), 3T images (*n = *33), and all images (*n = *60). Although 1.5T and 3T images have different signal-to-noise ratios, contrast-to-noise ratios, and magnetic field inhomogeneities, previous work has demonstrated a high correspondence between selected cortical, subcortical, and cerebrospinal fluid-filled spaces [40]. Pfefferbaum and colleagues found that the differences in structure volumes of 114 adults scanned at 1.5T and 3T field strengths within 3 weeks were generally close to the identity line, with some systematic differences slightly above and below depending on brain structure [40]. In addition, Kazemi et al. [41] combined 1.5T and 3T images for a neonate template (i.e., thirty 1.5T images and three 3T images). Based on evidence that the similarities will allow for successful combination of 1.5T and 3T datasets, we generated the combined template which increased the number of subjects contributing to the template yielding a more accurate representation of the true 6-month infant morphology.

Image intensity profile charts from each template were mapped along axes chosen for highly variable intensity patterns in order to qualitatively assess agreement. Convergence of the average head templates was quantitatively assessed by calculating the root mean square of voxel intensity differences (RMSd) between successive iterations. In addition, we took four measurements of physical dimensions: AC-PC length, head length (skull to skull at maximum left-right distance), breadth (skull to skull at maximum anterior to posterior distance), and height (maximum distance from the most superior point of the brain to the most inferior point of the temporal lobe). With these dimensions we compared our new average 6-month old head templates to the Sanchez et al. [25] average 6-month old templates, NIHPOD0-2 template, and the standard adult MNI152 template.

### Infant Atlas Creation

The 6 month old average template that was created using the procedure above was skull stripped to extract just the brain using the FSL Brain Extraction Tool (BET) [34], [42], followed by manual brain extraction. Manual clean up of the automated brain extraction results was performed by an operator with professional knowledge of neuroanatomy, removing any remaining non-brain structures by creating manually delineated brain masks to ensure only brain areas remained. Then, the extracted 6-month-old average brain was segmented into brain tissue and cerebral spinal fluid using FSL FMRIB’s Automated Segmentation Tool (FAST) [34], [43].

Previous work has employed various approaches to infant atlas generation. For example, Shi et al. [44] used a group average of atlases from 95 individuals, and Gousias et al. [45] used automatic segmentation from a group. Our method for creating the anatomic atlas for the 6 month head template involved several steps. First, the Anatomical Automatic Labeling (AAL) atlas [46] was coregistered to the 6-month-old average brain using MRIcro’s [47] CH2bet structural image [48] and the skull stripped 6-month-old average brain as inputs to FSL FMRIB’s Linear Image Registration Tool (FLIRT) [34], [49], [50]. The affine transformation matrix resulting from this coregistration was then applied to the AAL atlas to transform it to the 6-month-old average brain space. This coregistration result was the starting point for the final atlas. The resulting AAL to infant brain atlas was then adjusted by dilating and eroding individual masks of the anatomical regions using FSL’s mathematical manipulation tool fslmaths [34], followed by slice-by-slice manual adjustments of each region by an expert. The masks, created from specific infant templates, included the Sylvian fissure, the central fissure, the calcarine fissure, the internal capsule, the corpus callosum, the outer cerebrum boundary, the cerebrum midline boundary, cerebellum/cerebrum boundary, and the outer cerebellum boundary.

## Results and Discussion

**Figure 2:**
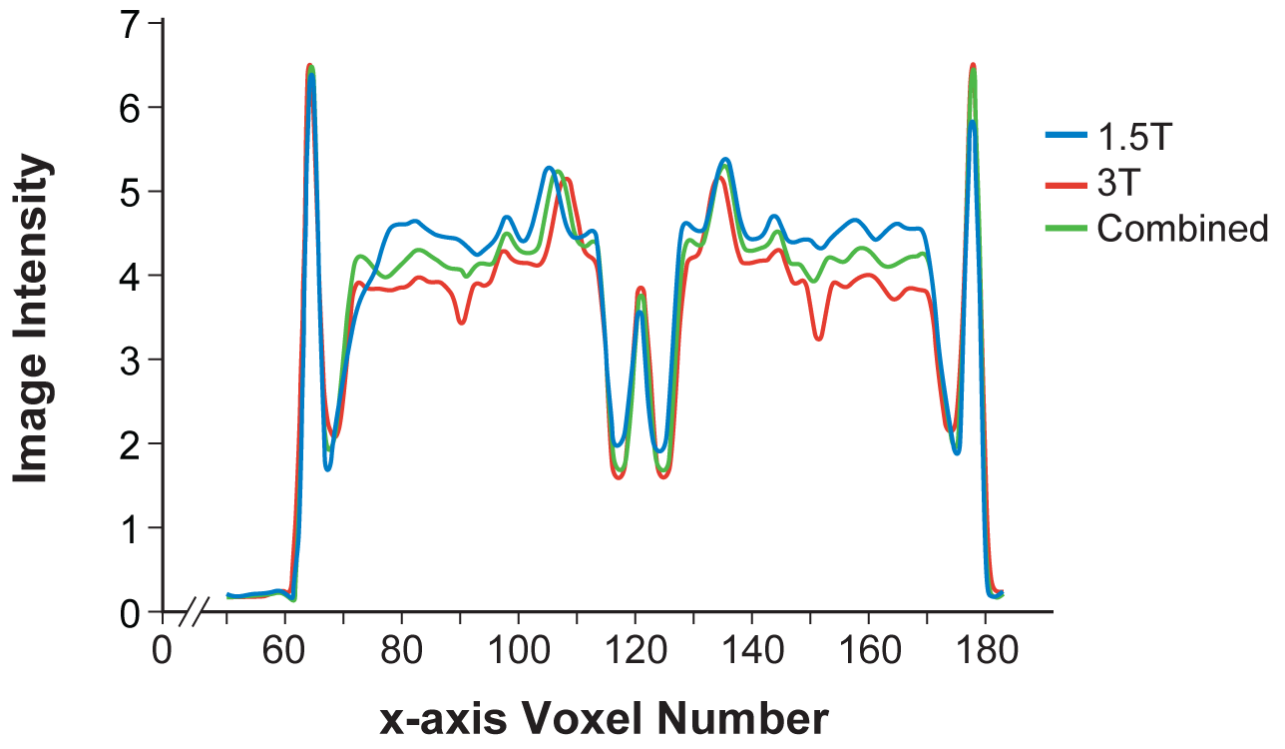
Representative intensity profile from a location including gray matter, white matter, and cerebrospinal fluid. Image intensity profile chart along the x axis at y = 141 and z = 79 showing intensity profiles for all three templates.

**Figure 3:**
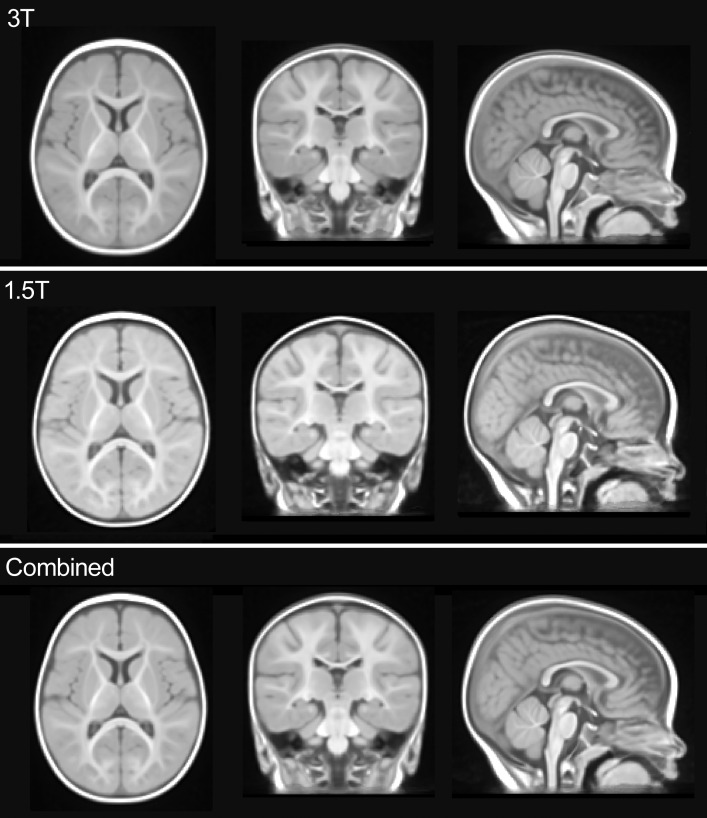
Average head templates. A qualitative comparison of the 3T age-specific 6-month-old infant average *(n = *33), the 1.5T age-specific 6-month-old infant average (*n = *27), and the combined age-specific 6-month-old infant average (*n = *60). Sagittal, coronal and axial slices are shown at (97, 116, 79).

**Figure 4:**
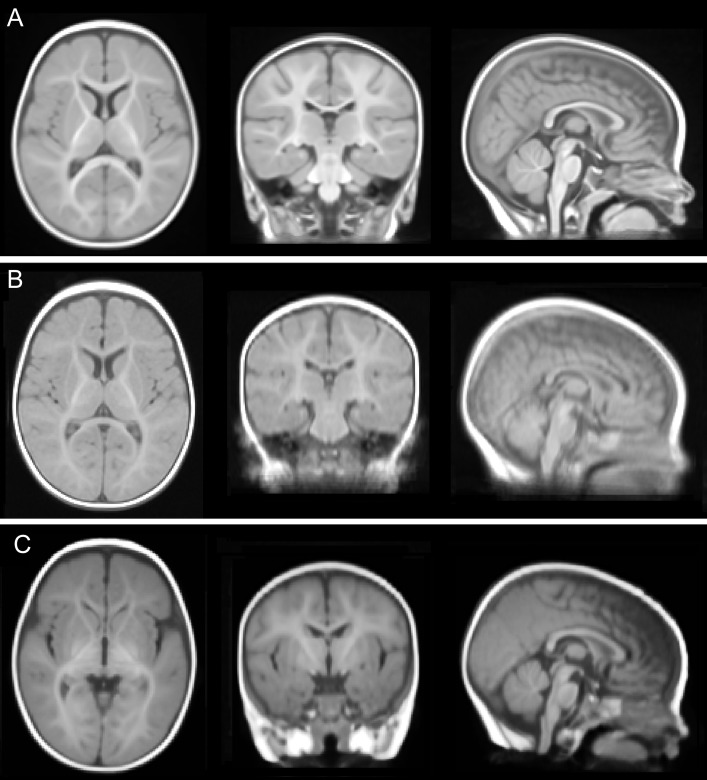
Comparison of average head templates. A qualitative comparison of (A) the new combined age-specific 6-month-old infant average template, (B) Fonov et al. NIHPD5–8m template for ages 5–8 months [2] and (C) Sanchez et al. 6-month-old average template [25]. Sagittal, coronal and axial slices are shown at (97, 116, 79). Not to scale, see Table 2 for measurements.

Unbiased, optimal average head templates were created from 1.5T images (N = 27), 3T images (N = 33), and all images (N = 60). These templates are publicly available from our website, http://ilabs.washington.edu/6-m-templates-atlas. A representative intensity profile taken along the x axis at y = 141 and z = 79, a location that includes gray matter, white matter, and cerebrospinal fluid, shows strong similarity between all three templates (Figure 2). Our three new average head templates (Figure 3) preserved the actual head size of 6-month-old infant subjects and were comparable in anatomic detail, sharpness and clarity. Our templates (represented by the combined template in Figure 4A) contained significantly more anatomical detail, sharpness, and clarity in both the brain and non-brain parts when compared to the NIHPD5–8m average template (Figure 4B). In addition, we compared our templates to a 6-month-old average template by Sanchez et al. [25] (Figure 4C), created by averaging 42 subjects (32 subjects from the NIHPD and 10 3T 3D MR images with voxel size 1×1×1 mm), employing a similar registration and iterative averaging method. Again, our templates exhibit much sharper and clearer composition of the head, similar to the comparison with the NIHPD5–8m template. The differences in the clarity among the templates are most likely due to the spatial resolution and quality of the input MR images (e.g., scan time, pulse sequence, motion during scan). Our template compares in clarity and composition to the 3T average template created by Sanchez et al. averaging 10 MR images [25]. Moreover, our average templates have successfully been created in the standard iMNI infant coordinate system proposed by Fonov et al. [2]. As a result, our new average head templates may be better suited as standard reference templates for the 6-month-old infant population when performing various neuroimaging analysis procedures.

**Figure 5:**
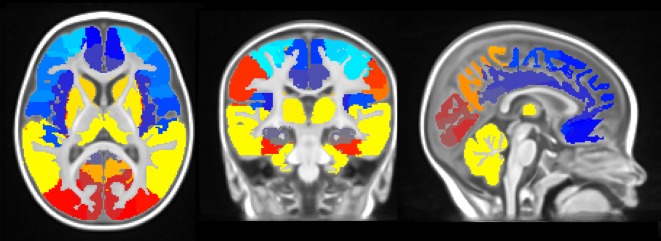
Six-month-old infant atlas. The color overlay of the AAL, which has been carefully modified to fit the average 6-month-old head morphometry, is superimposed on the 6-month-old average template. Sagittal, coronal and axial slices are shown at (97, 116, 79).

In Figure 5, a 6-month-old infant atlas that is compatible with the average template is shown overlaid on top of our combined template. The atlas contains 116 brain cortical regions. The axial view of the atlas in Figure 5 shows well-defined boundaries between frontal/temporal/occipital regions that match the template anatomy. The caudate (shown in yellow orange in Figure 5) also matches the template anatomy very closely. The atlas is publicly available from our website, http://ilabs.washington.edu/6-m-templates-atlas.

### Template Convergence

**Figure 6:**
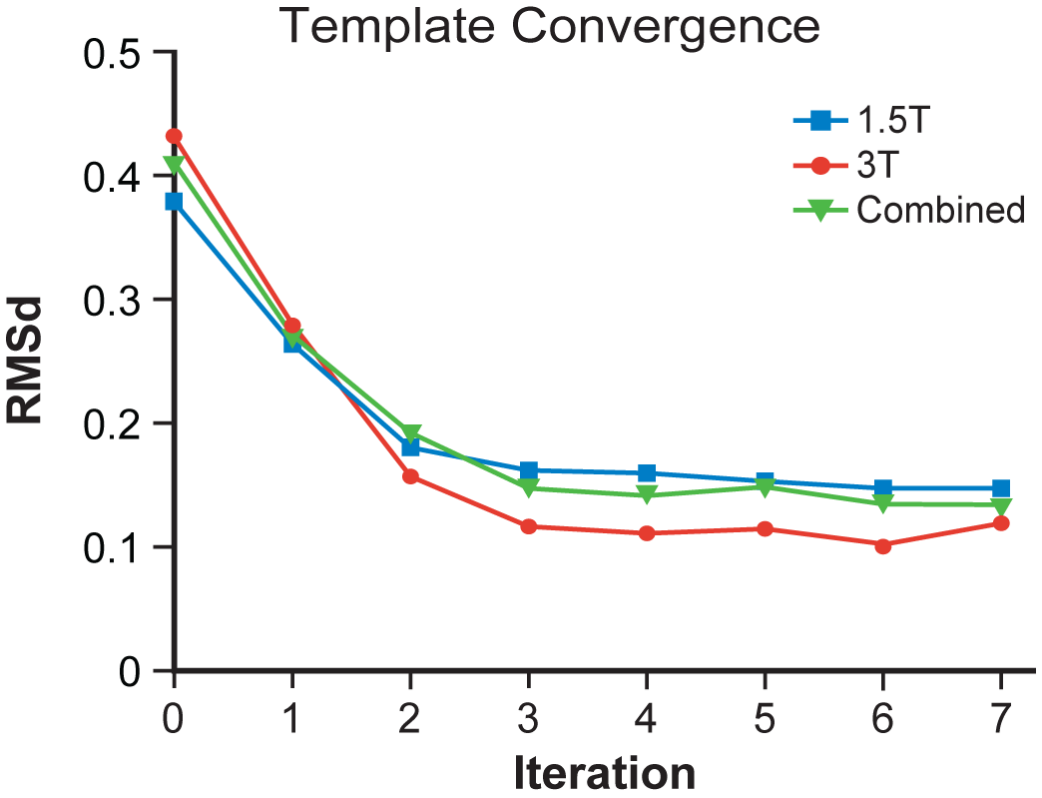
Template convergence. The RMSd between each average head template across successive iterations for generating the average head template. The RMSd exponentially decreased as the iterative averaging process proceeded. Convergence of the average head templates was reached by the fourth iteration.

Our new standard templates demonstrated similar trends of exponential increase in convergence as the iterative averaging process progressed. This resulted in the decrease of shape and appearance distances among all S_n,iMNI_ and the successive average templates (Figure 6). Convergence was confirmed by observing an exponential decrease in the RMSd values between successive iteration templates. The average templates reached convergence by the fourth iteration (*x* = 4), which is within the typical three to five iterations range that is known to be required for complete average template convergence [28].

### Global Shape Analysis of the Average Templates

Brain size is crucial in neural current localization using the MEG data because the distance between the magnetic sensors and the neural current location plays an important role in determining the neural current amplitude. Our method was designed to retain the 6-month-old infant brain size. Measurements of our proposed new templates, the NIHPD5–8m template, the Sanchez et al. [25] template, and the standard adult MNI152 template are compared in Table 2. All measurements from the average infant templates were 80% below the adult head measurements taken from the MNI152 template. Our new average templates and the Sanchez et al. [25] average template were very similar (ratio range = 0.70–74), whereas measurements from the NIHPD5–8m template were larger. Variation between the two average 6-month-old infant templates and the NIHPD5–8m template is reasonable since the NIHPD5–8m template used a 9-parameter linear registration to an adult ICBM template as the starting point, while our template used a 6-parameter rigid body registration to preserve the original size of the infant brain and head.

**Table 2:** Global shape analysis of the average templates.

	mm (ratio**)			
	AC-PC	length	breadth	height
Akiyama et al. (2013)*	19 (0.70)	150 (0.74)	122 (0.73)	97 (0.73)
Sanchez et al.	19 (0.70)	150 (0.74)	118 (0.71)	97 (0.73)
NIHPD5–8m	20 (0.74)	160 (0.78)	126 (0.75)	102 (0.77)
MNI152	27 (1)	203 (1)	167 (1)	132 (1)

* Measurements are identical for Akiyama et al. 1.5T, 3T, and combined templates.

** Ratio calculated based on corresponding measurement from MNI152 template.

### MEG Coordinate Space Transformation

**Figure 7:**
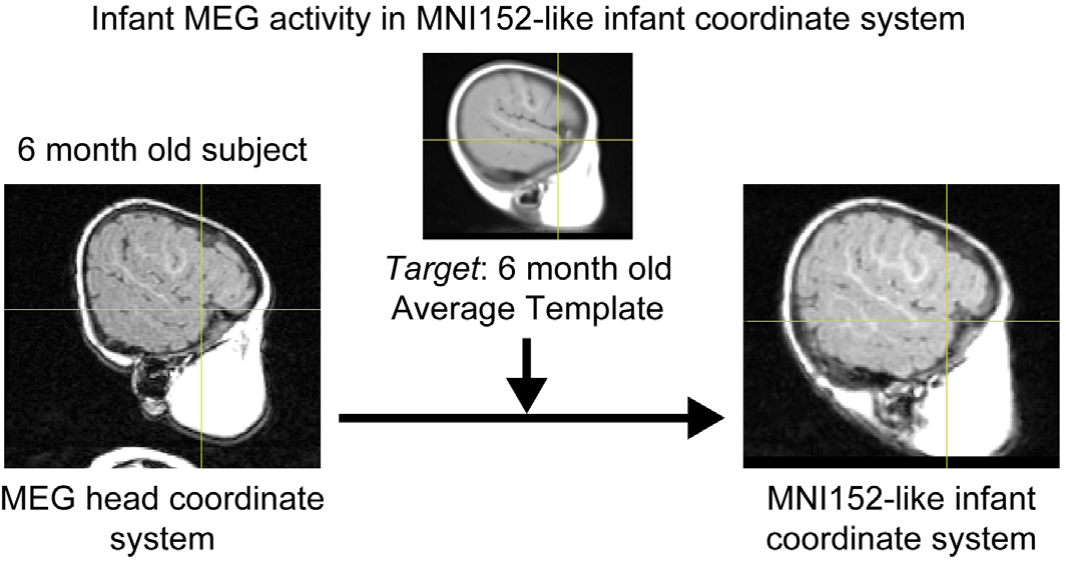
MEG coordinate space transformation. Estimated MEG activity location in a representative 6-month-old subject that has been successfully converted from the MEG subject-specific head coordinate system (left) to the standard iMNI infant coordinate system (right) by applying the appropriate transformation matrices.

A highlighted usage of these average infant templates is the transformation of estimated MEG activity locations expressed in MEG head coordinates into iMNI infant coordinates. This conversion is performed by applying the product of the rigid body transformation between subject-specific head coordinates and the raw subject scanner space coordinates, and the affine transformation between the raw subject scanner space coordinates and the iMNI infant coordinates using our newly created combined average template. Figure 7 shows the estimated MEG activity location in a representative 6-month-old subject, originally in Elekta-Neuromag (Elekta-Neuromag Oy, Helsinki, Finland) subject-specific head coordinates (left), and correctly converted to the iMNI infant coordinates by applying the appropriate transformation matrices (right). The estimated MEG activity locations are displayed within our new 6-month-old combined average template, which has been successfully converted to an Elekta-Neuromag-compatible FIF (functional image format) file. Furthermore, the iMNI infant coordinates can be transformed to the standard adult MNI152 coordinates by applying a constant scaling factor if necessary.

### Future usage for MEG Analysis

It is difficult to accomplish a group-level representation of infant MEG brain activation data employing individual tessellated brain meshes for each infant subject due wide variation in brain mesh shape and size. Our newly constructed age-specific average infant template provides an infant-specific iMNI standard coordinate system that will improve group level analysis. Furthermore, tessellated brain surface meshes for Boundary Element Model (BEM) from skull-stripped versions of these average templates can be constructed to calculate the neural currents.

## Conclusions

We provide new age- and population-specific unbiased average head templates and an atlas for typically developing, healthy 6-month-old infants that will be beneficial for structural and functional neuroimaging analyses of infant data. Our sample sizes are larger than other publicly available average infant templates at 6 months, making our template much more representative of this age-specific population [2], [25], [44]. In addition, our template exhibits high clarity and sharpness of the brain anatomy despite the large sample size. We have demonstrated an example usage of our template in accurately converting infant MEG activity locations from MEG subject-specific head coordinates to standard iMNI infant coordinates by applying the appropriate transformation matrices. We are continuing to increase the sample size for our age group to further enhance the population representation of our template and we will create a corresponding probabilistic atlas, and tessellated brain mesh. Furthermore, we anticipate additional applications of this template in other types of MEG analysis, such as BEM, and in other neuroimaging modalities.

## References

[1] Evans AC, Collins DL, Mills SR, Brown ED, Kelly RL, et al.. (1993) 3D statistical neuroanatomical models from 305 MRI volumes. Proc IEEE-NuclSciSymp Med Imaging Conf. pp. 1813–1817.

[2] Fonov VS (2009) Unbiased nonlinear average age-appropriate brain templates from birth to adulthood. NeuroImage 47: S102.

[3] Fonov V, Evans AC, Botteron K, Almli CR, McKinstry RC, et al (2011) Unbiased average age-appropriate atlases for pediatric studies. NeuroImage 54: 313–27. 20656036 10.1016/j.neuroimage.2010.07.033 PMC2962759

[4] Grabner G, Janke AL, Budge MM, Smith D, Pruessner J, et al (2006) Symmetric atlasing and model based segmentation: an application to the hippocampus in older adults. MICCAI 9: 58–66. 10.1007/11866763_8 17354756

[5] Mazziotta J (2001) A probabilistic atlas and reference system for the human brain. Philos T R Soc Lond B 35: 1293–1322. 10.1098/rstb.2001.0915 PMC1088516 11545704

[6] Mazziotta JC, Toga AW, Evans A, Fox P, Lancaster J (1995) A probabilistic atlas of the human brain: theory and rationale for its development. NeuroImage 2: 89–101. 9343592 10.1006/nimg.1995.1012

[7] Burgund ED, Kang HC, Kelly JE, Buckner RL, Snyder AZ, et al (2002) The feasibility of a common stereotactic space for children and adults in fMRI studies of development. NeuroImage 17: 184–200. 12482076 10.1006/nimg.2002.1174

[8] Hoeksma MR, Kenemans JL, Kemner C, Van Engeland H (2005) Variability in spatial normalization of pediatric and adult brain images. Clin Neurophysiol 116: 1188–1194. 15826861 10.1016/j.clinph.2004.12.021

[9] Murgasova M, Dyet L, Edwards D, Rutherford M, Hajnal JV, et al (2007) Segmentation of brain MRI in young children. Acad Radiol 14: 1350–1366. 17964459 10.1016/j.acra.2007.07.020

[10] Muzik O, Chugani DC, Juhász C, Shen C, Chugani HT (2000) Statistical parametric mapping: assessment of application in children. NeuroImage 12: 538–549. 11034861 10.1006/nimg.2000.0651

[11] Wilke M, Holland SK (2003) Variability of gray and white matter during normal development: a voxel-based MRI analysis. Neuroreport 14: 1887–1890. 14561914 10.1097/01.wnr.0000090951.15465.c8 PMC2268733

[12] Wilke M, Schmithorst VJ, Holland SK (2002) Assessment of spatial normalization of whole-brain magnetic resonance images in children. Hum Brain Mapp 17: 48–60. 12203688 10.1002/hbm.10053 PMC6871874

[13] Yoon U, Fonov VS, Evans AC, Perusse D (2009) The effect of template choice on morphometric analysis of pediatric brain data. NeuroImage 45: 769–777. 19167509 10.1016/j.neuroimage.2008.12.046

[14] Gaillard WD (2001) Developmental aspects of pediatric fMRI: considerations for image acquisition, analysis and interpretation. NeuroImage 13: 239–249. 11162265 10.1006/nimg.2000.0681

[15] Kochunov P, Lancaster JL, Thompson P, Woods R, Mazziotta J, et al (2001) Regional spatial normalization: toward an optimal target. J Comput Assist Tomogr 25: 805–816. 11584245 10.1097/00004728-200109000-00023

[16] Kochunov P, Lancaster JL, Hardies J, Thompson PM, Woods RP, et al (2005) Mapping structural differences of the corpus callosum in individuals with 18q deletions using targetless regional spatial normalization. Hum Brain Mapp 24: 325–331. 15704090 10.1002/hbm.20090 PMC6871744

[17] Leow AD, Klunder AD, Jack CR, Toga AW, Dale AM, et al (2006) Longitudinal stability of MRI for mapping brain change using tensor-based morphometry. NeuroImage 31: 627–640. 16480900 10.1016/j.neuroimage.2005.12.013 PMC1941663

[18] Senjem ML, Gunter JL, Shiung MM, Petersen RC, Jack CR (2005) Comparison of different methodological implementations of voxel-based morphometry in neurodegenerative disease. NeuroImage 26: 600–608. 15907317 10.1016/j.neuroimage.2005.02.005 PMC2739382

[19] Woods RP (2003) Characterizing volume and surface deformations in an atlas framework: theory, applications, and implementation. NeuroImage 18: 769–788. 12667854 10.1016/s1053-8119(03)00019-3

[20] Matsuzawa J, Matsui M, Konishi T, Noguchi K, Gur RC, et al (2001) Age-related volumetric changes of brain gray and white matter in healthy infants and children. Cereb Cortex 11: 335–42. 11278196 10.1093/cercor/11.4.335

[21] Knickmeyer RC, Evans D, Hamer RM, Gilmore JH, Gouttard S, et al (2008) A structural MRI study of human brain development from birth to 2 years. J Neurosci 28: 12176–12182. 19020011 10.1523/JNEUROSCI.3479-08.2008 PMC2884385

[22] Choe M, Ortiz-Mantilla S, Makris N, Gregas M, Bacic J, et al.. (2012) Regional Infant Brain Development: An MRI Based Morphometric Analysis in 3 to 13 Month Olds. Cereb Cortex 3. 10.1093/cercor/bhs197 PMC3729199 22772652

[23] Almli CR, Rivkin MJ, McKinstry RC (2007) The NIH MRI study of normal brain development (objective-2): Newborns, infants, toddlers, and preschoolers. NeuroImage 35: 308–325. 17239623 10.1016/j.neuroimage.2006.08.058

[24] Brain Development Cooperative Group (2006) The NIH MRI study of normal brain development. NeuroImage 30: 184–202. 16376577 10.1016/j.neuroimage.2005.09.068

[25] Sanchez CE, Richards JE, Almli CR (2012) Neurodevelopmental MRI brain templates for children from 2 weeks to 4 years of age. Developmental Psychobiology 54: 77–91. 21688258 10.1002/dev.20579 PMC3184192

[26] Avants BB (2004) Geodesic estimation for large deformation anatomical shape averaging and interpolation. NeuroImage 23: S139–S150. 15501083 10.1016/j.neuroimage.2004.07.010

[27] Avants BB (2008) Symmetric diffeomorphic image registration with cross-correlation: Evaluating automated labeling of elderly and neurodegenerative brain. Med Image Anal 12: 26–41. 17659998 10.1016/j.media.2007.06.004 PMC2276735

[28] Avants BB (2011) A reproducible evaluation of ANTs similarity metric performance in brain image registration. NeuroImage 54: 2033–2044. 20851191 10.1016/j.neuroimage.2010.09.025 PMC3065962

[29] Guimond A, Meunier J, Thirion JP (1998) Automatic computation of average brain models. MICCAI: Lecture Notes in Computer Science 1496: 631–640.

[30] Guimond A, Meunier J, Thirion JP (2000) Average brain models: a convergence study. Comput Vis Image Underst 77: 192–210.

[31] Sled JG (1998) A nonparametric method for automatic correction of intensity nonuniformity in MRI data. IEEE TMI 17: 87–97. 10.1109/42.668698 9617910

[32] MINC. Available: http://www.bic.mni.mcgill.ca/ServicesSoftware. Accessed 2012 Aug 23.

[33] McAuliffe M (2001) Medical image processing, analysis and visualization in clinical research. Proc IEEE CBMS. pp. 381–386.

[34] Smith SM, Jenkinson M, Woolrich MW, Beckmann CF, Behrens TEJ, et al (2004) Advances in functional and structural MR image analysis and implementation as FSL. NeuroImage 23: 208–219. 10.1016/j.neuroimage.2004.07.051 15501092

[35] Ad-Dab’bagh Y, Einarson D, Lyttelton O, Muehlboeck JS, Mok K, et al. (2006) The CIVET image-processing environment: a fully automated comprehensive pipeline for anatomical neuroimaging research. Poster presented at the 12^th^ Annual Meeting of the Organization for Human Mapping, Florence, Italy.

[36] Collins DL, Neelin P, Peters TM, Evans AC (1994) Automatic 3D Inter-Subject Registration of MR Volumetric Data in Standardized Talairach Space. J Comput Assist Tomogr 18: 192–205. 8126267

[37] Klein A, Andersson J, Ardekani BA, Ashburner J, Avants BB, et al (2009) Evaluation of 14 nonlinear deformation algorithms applied to human brain MRI registration. NeuroImage 46: 786–802. 19195496 10.1016/j.neuroimage.2008.12.037 PMC2747506

[38] Nyul LG (1999) On standardizing the MR image intensity scale. Magn Reson Med 42: 1072–1081. 10571928 10.1002/(sici)1522-2594(199912)42:6<1072::aid-mrm11>3.0.co;2-m

[39] Klein A, Ghosh SS, Avants BB, Yeo BTT, Fischl B, et al (2010) Evaluation of volume-based and surface-based brain image registration methods. NeuroImage 51: 214–220. 20123029 10.1016/j.neuroimage.2010.01.091 PMC2862732

[40] Pfefferbaum A, Rohlfing T, Rosenbloom MJ, Sullivan E (2012) Combining atlas-based parcellation of regional brain data acquired across scanners at 1.5 T and 3.0 T field strengths. NeuroImage 60: 940–951. 22297204 10.1016/j.neuroimage.2012.01.092 PMC3303927

[41] Kazemi K, Moghaddam HA, Grebe R, Gondry-Jouet C, Wallois F (2007) A neonatal atlas template for spatial normalization of whole-brain magnetic resonance images of newborns: Preliminary results. NeuroImage 37: 463–473. 17560795 10.1016/j.neuroimage.2007.05.004

[42] Smith SM (2002) Fast robust automated brain extraction. Hum Brain Mapp 17: 143–155. 12391568 10.1002/hbm.10062 PMC6871816

[43] Zhang Y, Brady M, Smith S (2001) Segmentation of brain MR images through a hidden Markov random field model and the expectation maximization algorithm. IEEE Trans. on Medical Imaging 20: 45–57. 11293691 10.1109/42.906424

[44] Shi F, Yap PT, Wu G, Jia H, Gilmore JH, et al (2011) Infant brain atlases from neonates to 1- and 2-year-olds. Plos One 6: e18746. 21533194 10.1371/journal.pone.0018746 PMC3077403

[45] Gousias IS, Rueckert D, Heckemann RA, Dyet LE, Boardman JP, et al (2008) Automatic segmentation of brain MRIs of 2-year-olds into 83 regions of interest. NeuroImage 40: 672–84. 18234511 10.1016/j.neuroimage.2007.11.034

[46] Tzourio-Mazoyer N, Landeau B, Papathanassiou D, Crivello F, Etard O, et al (2002) Automated anatomical labelling of activations in spm using a macroscopic anatomical parcellation of the MNI MRI single subject brain. NeuroImage 15: 273–289. 11771995 10.1006/nimg.2001.0978

[47] Rorden C, Brett M (2000) Stereotaxic display of brain lesions. Behav Neurol 12: 191–200. 11568431 10.1155/2000/421719

[48] Holmes CJ, Hoge R, Collins L, Woods R, Toga AW, et al (1998) Enhancement of MR images using registration for signal averaging. J Comput Assist Tomogr 22: 324–33. 9530404 10.1097/00004728-199803000-00032

[49] Jenkinson M, Smith SM (2001) A global optimisation method for robust affine registration of brain images. Med Image Anal 5: 143–156. 11516708 10.1016/s1361-8415(01)00036-6

[50] Jenkinson M, Bannister PR, Brady JM, Smith SM (2002) Improved optimisation for the robust and accurate linear registration and motion correction of brain images. NeuroImage 17: 825–841. 12377157 10.1016/s1053-8119(02)91132-8

